# Patterns of species diversity and phylogenetic structure of vascular plants on the Qinghai-Tibetan Plateau

**DOI:** 10.1002/ece3.847

**Published:** 2013-10-21

**Authors:** Yujing Yan, Xian Yang, Zhiyao Tang

**Affiliations:** Department of Ecology, College of Urban and Environmental Sciences and Key Laboratory for Earth Surface Processes, Peking UniversityBeijing, 100871, China

**Keywords:** Climate, pattern, phylogenetic structure, Qinghai-Tibetan Plateau, species richness, vascular plants

## Abstract

Large-scale patterns of species richness and the underlying mechanisms regulating these patterns have long been the central issues in biogeography and macroecology. Phylogenetic community structure is a result of combined effects of contemporary ecological interactions, environmental filtering, and evolutionary history, and it links community ecology with biogeography and trait evolution. The Qinghai-Tibetan Plateau provides a good opportunity to test the influence of contemporary climate on shaping species richness because of its unique geological history, cold climate, and high biodiversity. In this study, based on high-resolution distributions of *˜*9000 vascular plant species, we explored how species richness and phylogenetic structure of vascular plants correlate with climates on the highest (and species rich) plateau on the Earth. The results showed that most of the vascular plants were distributed on the eastern part of the plateau; there was a strong association between species richness and climate, even after the effects of habitat heterogeneity were controlled. However, the responses of richness to climate remarkably depended on life-forms. Richness of woody plants showed stronger climatic associations than that of herbaceous plants; energy and water availability together regulated richness pattern of woody plants; whereas water availability predominantly regulated richness pattern of herbaceous plants. The phylogenetic structure of vascular species clustered in most areas of the plateau, suggesting that rapid speciation and environment filtering dominated the assembly of communities on the plateau. We further propose that biodiversity conservation in this area should better take into account ecological features for different life-forms and phylogenetic lineages.

## Introduction

Patterns and the underlying mechanisms of species richness are among the central issues in ecology and biogeography (Rosenzweig 1995; Ricklefs 2003; Willig et al. 2003; Buckley et al. 2010; Jetz and Fine 2012). Numerous hypotheses have been proposed to explain the geographical patterns of species richness (Willig et al. 2003; Mittelbach et al. 2007). These hypotheses can be grouped into two categories: one argues that contemporary environments, such as climate and habitat heterogeneity, regulate the current patterns of biodiversity (Kerr and Packer 1997; Brown et al. 2004; Currie et al. 2004; Wang et al. 2009b), the second hypothesizes that historical processes, such as speciation, extinction, and dispersal, predominantly control the current patterns of biodiversity (Ricklefs 1987, 2003, 2005; Zobel 1997; Mittelbach et al. 2007). Although no consensus has been reached on the relative roles of contemporary environments and historical processes on diversity patterns, it is widely accepted that these factors jointly regulated these patterns (Hawkins and Porter 2003; Svenning and Skov 2005, 2007; Montoya et al. 2007; Wang et al. 2012). However, it is difficult to disentangle the independent effects of historical processes and contemporary environment because of the colinearity between these two factors, for example, different regions (potentially underwent different historical processes) normally have different climates (Ricklefs 2005; Svenning and Skov 2005; Wang et al. 2012).

The analysis of community phylogenetic structure can help reveal the ecological and evolutionary processes regulating coexistence of species at different scales (Webb et al. 2008; Vamosi et al. 2009; Jetz et al. 2012). The relatedness of species occurring in a community represents the balance of different ecological and evolutionary processes that determine their distribution and abundance (Webb et al. 2002). Nonrandom phylogenetic structure of communities at large-scales is the result of overlap of species ranges caused by the joint effect of macroevolutionary (speciation or extinction) and ecological processes (competitive exclusion, environmental filtering, and dispersal) (Cavender-Bares et al. 2006; Kraft et al. 2007; Webb et al. 2008; Gotelli et al. 2009; Cardillo 2011; Villalobos et al. 2013). It reflects the community assemblages in relative complete areas of species distribution (Villalobos et al. 2013). Specifically, phylogenetic clustering in a region indicates that environments act as a filter to select for species in possession of certain traits, or the region underwent rapid speciation rates or slow extinction rates (Wiens and Graham 2005; Cardillo 2011);, whereas phylogenetic dispersion at regional scale indicates that niche evolution and the pervasion of competition exclusion may dominate the community assemblage (Burns and Strauss 2011; Villalobos et al. 2013).

The Qinghai-Tibetan Plateau is the highest plateau and one of the largest plateaus on the Earth with an average altitude of >4000 m and occupying ∼2.5 million km^2^ (Zhang et al. 2002). The following characteristics make the Qinghai-Tibetan Plateau a unique opportunity to test the effect of environments on the patterns of species richness. First, the plate collision-induced uplifting of the Qinghai-Tibetan Plateau was relatively rapid and uniform, especially in the Quaternary period; therefore, geological or historical processes are more or less consistent across the plateau and it is easy to distinguish the plateau from its nearby regions clearly (Zhang et al. 2002); the rapid uplift of the Qinghai-Tibetan Plateau has resulted in rapid and continuous specification and diversification for plants (Mao et al. 2010; Jia et al. 2012). Second, being a center of biodiversity hotspots (Tang et al. 2006; López-Pujol et al. 2011), the Qinghai-Tibetan Plateau provides habitats for ∼9000 vascular plants, of which ∼18% are endemic to this area (Wu 2008); the heterogeneous environments also depict that species richness varies remarkably among different areas across the plateau (Tang et al. 2006; Yang et al. 2013). Third, known as the third pole, the Qinghai-Tibetan Plateau is peculiarly cold for its latitude (Liu and Chen 2000); although most studies conducted in the high-latitude cold areas have concluded that temperature was the predominant factor of richness patterns (Danell et al. 1996; Hawkins and Porter 2003; Currie et al. 2004), how climate affects richness patterns in cold but relatively low latitudes is still unclear. Fourth, human activities are comparably slight compared with other regions (Bai and Zhang 2002; Cui and Graf 2009); therefore, lives on the plateau have not been so much influenced by human activities. Furthermore, in recent global studies on mapping diversity for vascular plants, the data quality and suitability in the Qinghai-Tibetan Plateau remain “poor” (Kier et al. 2005; Kreft and Jetz 2007); knowledge on the patterns and causes of species richness on this plateau is very limited (but see Mao et al. 2013). Long-term comprehensive investigation resulted in the completeness of specimen collection in this region (Sun 2007; Yang et al. 2013). As a result, several monographs on the distribution of vascular plants on this plateau have been published (Wu and Mei 2001; Wu 2008), providing an opportunity to explore the patterns of species richness on this plateau.

In this study, we exploited this opportunity to explore the effects of contemporary climate on the patterns of species richness and phylogenetic diversity of vascular plants on the extremely high and species-rich Qinghai-Tibetan Plateau. The questions we asked are (1) how species richness and phylogenetic diversity of vascular plants change along geographical and climatic gradients on the Qinghai-Tibetan Plateau and (2) which climatic variable(s) plays more roles in shaping these patterns and how. As the ecosystems in the Qinghai-Tibetan Plateau are highly vulnerable to rapid global climate change, studies on the species richness patterns in this high region are necessary for biodiversity conservation in a changing climate (Beniston 2003; Song et al. 2005).

## Data and Methods

### Distribution of vascular plants

The species distribution maps were compiled follow the monographs, *The vascular plants and their eco-geographical distribution of the Qinghai-Tibetan Plateau* (Wu 2008) and *The plant resources in source area of Yellow River and its environment* (Wu and Mei 2001). These checklists and distribution data were based on inventories conducted by dozens of “national teams” and hundreds of “local teams” for the plants and their distributions on the Qinghai-Tibetan Plateau during the past 60 years (Sun 2007; Wu 2008). The nomenclature followed the *Flora Republicae Popularis Sinicae* (Editorial Board of Flora Republicae Popularis Sinicae 1952–2004). We also referred to specimen record (http://www.cvh.org.cn/cms/), checklists of nature reserves, scientific publications, related provincial, and many local floras across this region to make the database more precise. A recent evaluation of the completeness of the specimen collection depicted that nearly all counties on the Qinghai-Tibetan Plateau has been completely investigated (Yang et al. 2013). We then checked the list with the APG III classification (The Angiosperm Phylogeny Group, 2009).

For each species, the distribution is recorded at two domains: horizontal distribution at county level and elevation range (the upper and lower elevation). The Qinghai-Tibetan Plateau includes 139 counties, and the areas of them varied remarkably. We then converted the distribution map of each species as grid-based map to eliminate the potential influence of area on the estimation of species richness. To do this, we first divided the plateau into 930 girds of 0.5º *0.5º (equivalent to 50 × 50 km, varied between *ca* 55.5 × 43.2 km and ∼55.5 × 49.0 km on the plateau). We then overlapped this grid map with DEM (at 1 arc-second resolution) and administrative map of China to get the elevation range and county (or counties) included in each grid. A species recorded as present in the grid when both its horizontal (county) and vertical (elevation range) distribution was included in the grid. Some grids contained more than one county; therefore, conversion only based on horizontal distribution might enlarge the distribution range of the species. However, this bias has been minimized because of the following two reasons: First, the area of a grid is much less than that of a county (∼15%); second, we added an elevation range for each species. The species richness is defined as the number of species occurring in a given grid.

### Environmental factors

We used habitat heterogeneity and climate to explore the determinants of patterns of plant diversity on the Qinghai-Tibetan Plateau.

#### Habitat heterogeneity

**Figure 1:**
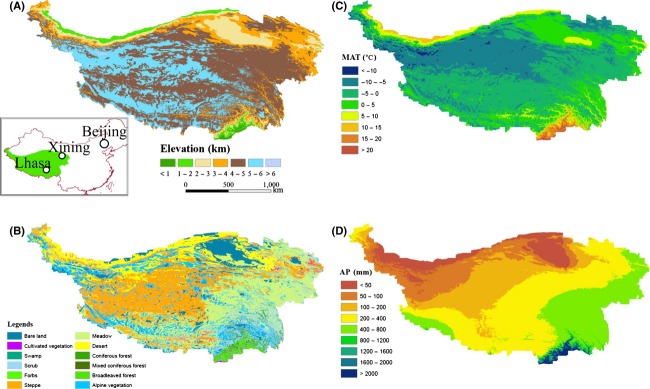
Distributions of (A) digital elevation model, (B) mean annual temperature, (C) annual precipitation, and (D) vegetation on the Qinghai-Tibetan Plateau. The subset figure in the (A) indicates the location of the Qinghai-Tibetan Plateau in China.

We used the following variables to represent habitat heterogeneity for each grid: elevation range (TOPO), the number (VT), and Shannon–Wiener index (SWV) of vegetation association types (association is defined as a group of plant communities sharing the same dominant species) in the Vegetation Map of China (Editorial Board of Vegetation map of China 2007) within the grid. TOPO was defined as the difference between the maximum and minimum elevations within a grid using a GTOPO30 digital elevation model (DEM) at 1-km resolution (available at http://eros.usgs.gov/products/elevation/gtopo30/gtopo30.html, Fig. 1A). VT was defined as the number of vegetation associations of a grid based on the 1:10,00,000 vegetation atlas of China (Editorial Board of Vegetation map of China 2007; Fig. 1B). SWV was calculated as:


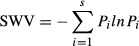


where *P*_*i*_ = (area of the _*i*_th vegetation association)/(total area of the grid).

#### Climate

Previous studies showed that energy, water availability, and climatic stability were regarded as important factors determining species diversity at large scales (Stevens 1989; Currie et al. 2004; Allen et al. 2007). In this study, we used mean annual temperature (MAT), mean temperature of the coldest (MTCM) and warmest month (MTWM), potential evapotranspiration (PET), coldness index (CI), and warmth index (WI) as surrogates of energy availability; annual precipitation (AP), actual evapotranspiration (AET), and annual rainfall (RAIN) as surrogates of water availability; and annual ranges of temperature (ART, defined as the difference between MTWM and MTCM), temperature and precipitation seasonality (TSN and PSN, the standard deviation of monthly mean temperature and precipitation) as surrogates of climatic stability. We extracted the average values of monthly temperature (MT) and precipitation (MP), AP, MAT, MTCM, MTWM, PSN, TSN, ART, and CI from the Worldclim spatial database at 30 arc-second resolution (Hijmans et al. 2005; available at http://www.worldclim.org/, Fig. 1C and D demonstrated the distribution of MAT and AP on the Qinghai-Tibetan Plateau) for each grid. Based on the MP and MT, we calculated PET and AET using the method proposed by Thornthwaite (1955) and WI as the sum of (MT-5) when MT > 5°C and RAIN as the sum of MP when MT > 0°C (Fang and Yoda 1990).

### Data analysis

#### Phylogenetic diversity and structure

We first constructed phylogenies of all species based on the R20120829 megatree in Phylomatic v3 (http://phylodiversity.net/phylomatic/). The branch length was assigned based on the divergence times estimated by Wikstrom et al. (2001). We used Faith's (1992) index, which is defined as the total branch length among all taxa in a grid, to measure the phylogenetic diversity (PD). As phylogenetic diversity was highly correlated with species richness (*R*^2^ = 0.96, 0.90, 0.84 for total diversity, woody, and herbaceous plants, respectively, all with *P* < 0.001), we therefore only reported the pattern of species richness.

We then calculated net relatedness index (NRI) to measure the phylogenetic structure. It is defined as the difference in mean phylogenetic distances (MPD) between observed and randomly generated null communities, standardized by the standard deviation of phylogenetic distances in the null communities (Webb et al. 2008), and calculated as:


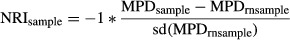


The null community containing the observed species richness in each site was randomly assembled from the species pool found in our study using the phylogeny shuffle null model; it shuffles species labels across the entire phylogeny with 999 iterations, thus randomizes phylogenetic relationships among species (Gotelli 2000). A positive NRI indicates phylogenetic clustering, whereas a negative NRI indicates phylogenetic overdispersion.

#### Statistic analysis

Spearman correlation was applied to explore the relationships between species richness and environmental variables (climate and habitat heterogeneity).

To eliminate the influence of collinearity between variables, partial redundancy analysis (partial RDA) was conducted to partition the effects of different explanatory variables (and/or combinations) using the R package Vegan (Oksanen et al. 2013). The total variance of species richness was partitioned into four parts: (i) and (ii) variance explained by the independent effect of either factor; (iii) variance explained by the covarying effect of both factors; and (iv) residuals. In this study, we first partitioned the effects of climate vs. habitation heterogeneity on species richness for different groups. To do this, we built three models to include the following predictors: (i) climate, (ii) habitat heterogeneity, and (iii) habitat heterogeneity plus climates. The same process was conducted using water vs. energy to partition the independent effects of water- and energy-related variables by building three models to include the following predictors: (i) energy-related variables, (ii) water-related variables, and (iii) energy-plus water-related variables. Finally, Monte Carlo permutation test was used to test the significance of each model. For detailed information on the partial ordination, see Borcard et al. (1992).

Net relatedness index exhibited different relationship at different parts along the environmental gradients. We then applied piecewise regressions using R package Segmented (Muggeo 2008) to find the breakpoints of the relationship between environmental variables and NRI of total diversity (Toms and Lesperance 2003). Piecewise linear regression attempts to find the heterogeneity of the relationship between two variables by identifying one or more breakpoints and then fits linear regressions for each piece before or after the breakpoints.

Spatial autocorrelation may inflate type I errors (Lennon 2000; Diniz-Filho et al. 2003). To evaluate the effect of spatial autocorrelation on the model, we calculated Moran's Index of spatial autocorrelation for the species richness and the residuals using the APE package in R (Paradis et al. 2004). The distances between the midpoints of grids were measured based on WGS84 spherical coordinates using R package geosphere (Hijmans et al. 2012). The results showed that species richness exhibited high spatial autocorrelation (Moran's *I* = 0.20, *P* < 0.01 in pattern of total diversity); the species richness–environment model (an OLS model with all environmental factors as independent variables, *R*^2^ = 0.54, *P* < 0.01) has remarkably decreased the spatial autocorrelation (Moran's Index = 0.05, *P* < 0.01 in pattern of residuals), indicating that the variables we chose have explained most of the spatial structure of the data. Therefore, we did not use the spatial linear autoregressive models (SLM), as the SLM may tend to underestimate the effects of predictors at large scales and this bias is hard to control (Diniz-Filho et al. 2003).

We conducted phylogenetic analysis using the Phylocom 4.2 software (Webb et al. 2008). Statistics analyses were conducted by RStudio 0.97.332 (RStudio 2012). Geostatistical processing was conducted using ArcGIS 9.3 (ESRI 2008).

## Results

### Geographical patterns of species richness, phylogenetic diversity and structure

In total, the database contained distributions of 8876 vascular species from 1371 genera and 211 families, including 6475 (72.9% of the total) herbaceous and 2401 (27.1%) woody plants, of which 1706 (19.2% of the total) were endemic to the plateau.

**Figure 2:**
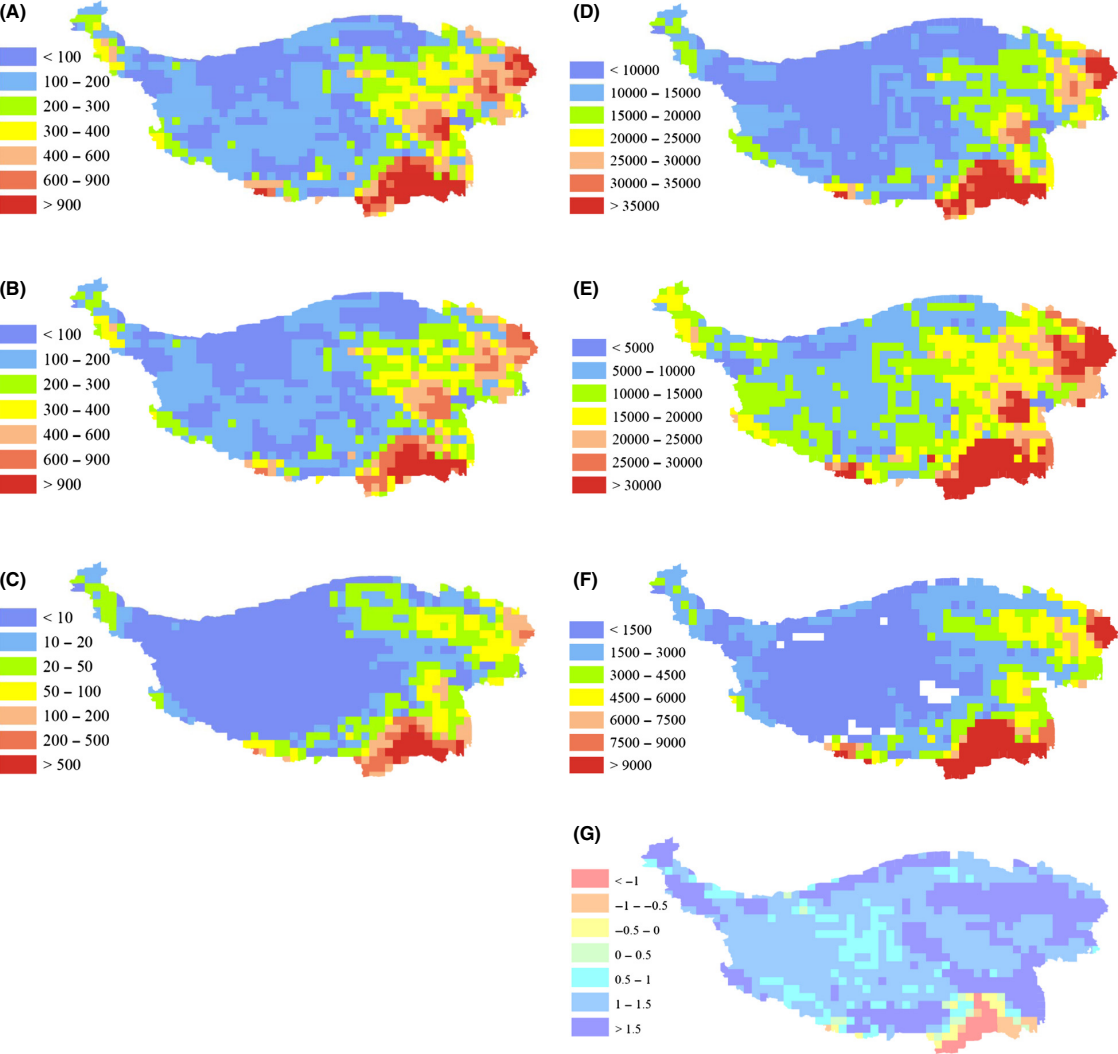
Geographical patterns of species richness and phylogenetic diversity (PD) for (A, D) total species, (B, E) herbaceous plants (C, F) woody plants, and (G) the phylogenetic structure (NRI) of total species on the Qinghai-Tibetan Plateau. Left column for species richness, and the right column for phylogenetic diversity (PD) and phylogenetic structure (NRI).

Plant species richness ranged from 1 to 3403 in each grid on the Qinghai-Tibetan Plateau, with an average of 328. Only five grids (0.5% of the total) have less than 10 species. Average richness of herbaceous and woody plants was 273 (1–2056) and 55 (0–1378), respectively. Species richness was higher in the mountains and valleys along the east and south margin of the plateau where the altitude is relatively low (<2000 m above sea level); and lower in the basins located on the northern part of plateau as well as the high-latitude (>4000 m above sea level) area in the central part of the plateau (Fig. 2A). Richness of herbaceous plants showed similar pattern as that of the total diversity (Fig. 2B) and that of woody plants was scattered in the southeastern and northeastern part of the plateau where it is relatively warm and wet (Fig. 2C), suggesting that the determinants of woody plant richness may be different from those of the total and herbaceous plants. Phylogenetic diversity showed the same spatial pattern as species richness of total (Fig. 2D), woody (Fig. 2E), and herbaceous plants (Fig. 2F).

Phylogenetic structure showed a different pattern compared with the species richness and phylogenetic diversity. NRI was positive all over the plateau excepting in the southeast, indicating that vascular plants on the Qinghai-Tibetan Plateau exhibited clustered phylogenetic structure. Highest NRI (>1.5) occurred in the eastern part as well as the edge of the plateau, whereas lowest NRI occurred in the southeast corner of the plateau (Fig. 2G).

### Relationships between species richness and environmental variables

Simple correlation indicated that coldness and precipitation were the main factors shaping the pattern of species richness for all groups. Water availability was the strongest predictor for the richness pattern of total vascular plants; it showed a correlation coefficient (R) of 0.64 with AP, 0.65 with AET and 0.63 with RAIN. The climatic correlates of richness were different between woody and herbaceous plants. CI (−0.55) and MAT (0.54) were the most significant predictors for species richness of woody plants, whereas AET (0.65), RAIN (0.63), and AP (0.64) were best for that of herbaceous plants. Habitat heterogeneity (VT) showed a correlation coefficient of 0.40 with richness of the total, 0.39 with that of herbaceous plants and 0.47 with that of woody plants (Table 1).

**Table 1:** Spearman correlation (R) between species richness, phylogenetic diversity, and environmental factors on the Qinghai-Tibetan Plateau

	Species richness			Phylogenetic diversity (PD)		
		
	All	Herbaceous	Woody	All	Herbaceous	Woody
Climate–Energy availability						
MAT	0.33	0.29	0.54	0.42	0.39	0.57
PET	0.27	0.23	0.48	0.35	0.31	0.51
MTCM	0.36	0.33	0.50	0.45	0.43	0.52
MTWM	0.23	0.19	0.47	0.31	0.27	0.51
CI	−0.36	−0.32	−0.55	−0.46	−0.43	−0.58
WI	0.24	0.20	0.49	0.32	0.29	0.52
Climate–Climatic stability						
ART	−0.41	−0.40	−0.30	−0.43	−0.44	−0.29
TSN	−0.42	−0.42	−0.31	−0.44	−0.46	−0.31
PSN	−0.40	−0.41	−0.32	−0.43	−0.44	−0.34
Climate–Water availability						
AP	0.64	0.64	0.49	0.69	0.70	0.52
AET	0.65	0.65	0.50	0.69	0.70	0.53
RAIN	0.63	0.63	0.50	0.68	0.70	0.53
Habitat Heterogeneity						
VT	0.40	0.39	0.47	0.45	0.43	0.54
SWV	0.38	0.37	0.44	0.42	0.40	0.49
TOPO	0.28	0.27	0.38	0.32	0.30	0.41

All with *P* value < 0.01.

MAT = mean annual temperature; PET = potential evapotranspiration; MTCM = mean temperature of the coldest month; MTWM = mean temperature of the warmest month; CI = coldness index; WI = warmth index; ART = annual range of temperature; TSN = temperature seasonality; PSN = precipitation seasonality; AP = annual precipitation; AET = actual annual evapotranspiration; RAIN = rainfall; VT = number of vegetation types; SWV = Shannon–Wiener Index of vegetation types; and TOPO = topographical heterogeneity.

**Figure 3:**
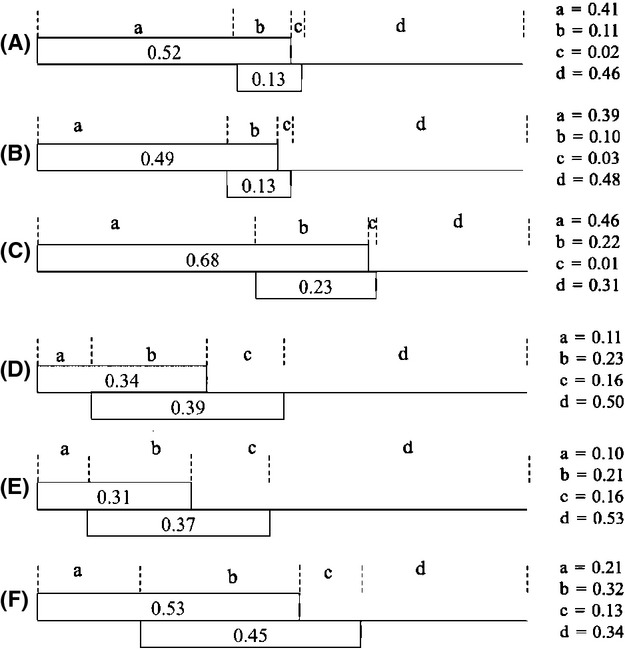
Comparisons of the effects of contemporary climate vs. habitat heterogeneity, as well as the effects of energy availability vs. water availability, on species richness for (A, D) total diversity, (B, E) herbaceous, and (C, F) woody plants on the Qinghai-Tibetan Plateau. In each subplot, the variation of species richness is partitioned into (a) the independent effect of climate, (b) the covarying effect, (c) the independent effect of habitat heterogeneity, and (d) residual variation.

Partial ordination indicated that climate and habitat heterogeneity together explained 54% of richness pattern for the total species, 69% for woody plants, and 52% for herbaceous plants, respectively. Climate independently explained 39–46% of the pattern, whereas habitat heterogeneity independently explained only 1–3% of the pattern. The joint effect of these two factors accounted for 10–22% of the pattern (Fig. 3). We further compared the relative importance of water availability and energy availability. Energy and water availability independently explained 11% and 16% of richness patterns of the total species, with 23% explained by their joint effects. For different life-forms, energy availability, water availability, and their joint effects explained 21%, 13%, and 32% of the variances for woody plant richness and 10%, 16%, and 21% of variance for herbaceous plant richness, respectively (Fig. 3).

### Relationships between phylogenetic structure and environmental variables

**Figure 4:**
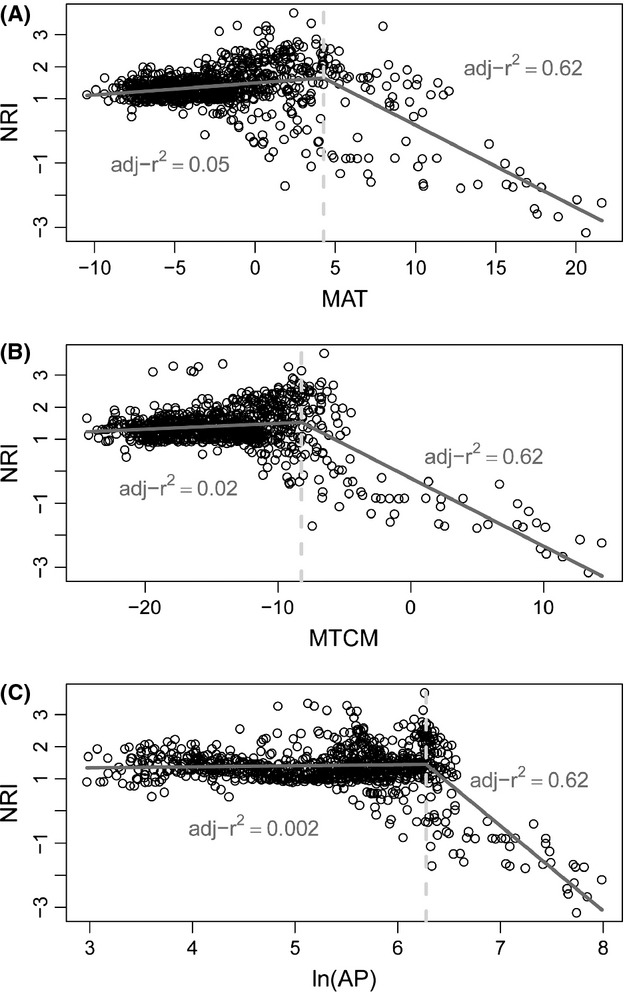
Changes in phylogenetic structure (NRI) along the gradients of (A) mean annual temperature (MAT, °C), (B) mean temperature of the coldest month (MTCM, °C), and (C) logarithm-transferred annual precipitation (AP, mm). The R^2^ of the piecewise regression was 0.35, 0.47, and 0.44, and breakpoint was 4.27°C, −8.24°C, and 6.28 for the MAT, MTCM, and logarithm-transferred AP (equivalent to AP = 532.7 mm), respectively.

Temperature and precipitation both strongly influenced the distribution of NRI of vascular plants on the Qinghai-Tibetan Plateau. The piecewise regression identified a threshold of 4.27°C along the MTCM gradient. The NRI kept almost stable when the MAT below 4.27°C (*R*^2^ = 5%), and it then decreased linearly as MAT increased (*R*^2^ = 62%, Fig. 4A). A piecewise regression indicated that MAT explained 35% of the variance in NRI. It showed the same patterns along the MTCM and AP gradients, with breakpoints at MTCM = −8.24°C (Fig. 4B, *R*^2^ = 2% and 62% when MTCM below and above −8.24°C; *R*^2^ = 47% for the piecewise repression) and AP = 532.7 mm (Fig. 4C, *R*^2^ = 0.2% and 62% when AP below and above 532.7 mm; *R*^2^ = 44% for the piecewise repression).

## Discussion

### Patterns of plant species diversity on the Qinghai-Tibetan Plateau

Using the distribution of ∼9000 vascular plants on the Qinghai-Tibetan Plateau, we explored large-scale patterns and effects of the contemporary factors determining these patterns of richness and phylogeny of vascular plant on the highest plateau on the Earth. Consistent with previous studies (Currie et al. 2004; Kreft and Jetz 2007; Wang et al. 2009b, 2011, 2012), we observed strong influences of climate on species richness patterns of vascular plants on the Qinghai-Tibetan Plateau, and this effect held when the effect of habitat heterogeneity was controlled. However, compared with similar studies in other areas (Wang et al. 2011), the association between species and climate is relatively weak, partly because the Qinghai-Tibetan Plateau is still uplifting (Zhang et al. 2002), many species are still in dispersing to their suitable habitats, and many new species are emerging (Xu et al. 2010). Specifically, inconsistent with the findings that species richness was determined by energy availability in cold regions at high latitude (e.g., Hawkins et al. 2003), water availability (such as AP, AET, and RAIN) primarily determines patterns of vascular plant richness on the Qinghai-Tibetan Plateau. This result reveals that the restriction of energy is not as important as we conceded before. The predominant effects of water inputs may indicate that the plateau is more arid than cold for the vascular plants. Several reasons may cause such climatic correlates with species richness. First, the Qinghai-Tibetan Plateau is comparably dry, with an annual precipitation of 296 mm; furthermore, the extreme high altitude brought about the high level of ultraviolet radiation associated with strong winds, which led to the high level of evaporation (Zhang et al. 2011). Other potential reasons include the small fraction of woody plants on the Qinghai-Tibetan Plateau (2401 of 8876 species, or 27.1% of the total); it is recognized that association between species richness and energy availability is stronger for woody than for herbaceous plants (Wang et al. 2009a; Hawkins et al. 2011; Axmanová et al. 2012; Reich et al. 2012).

Indeed, we observed different responses of species richness to climate between woody and herbaceous plants. These differences lie in two aspects: the predominant climatic factors and the strength of the association between richness and climate. Consistent with previous studies concerning woody plants across China (Wang et al. 2011), winter coldness (CI and MTCM) is the most important factor, but energy and water availability jointly regulate the patterns of woody species richness on the Qinghai-Tibetan Plateau. In contrast, richness of herbaceous plants is mostly regulated by water availability. The association between species richness and climate is stronger for woody plants than for herbaceous plants. The following reasons could provide some implications about the mechanisms of this difference. First, in addition to macroclimates, microenvironments such as light availability, soil pH, and nutrient availability may strongly affect richness of herbaceous plants (Oberle et al. 2009; Wang et al. 2009a; Axmanová et al. 2012; Reich et al. 2012); Second, herbaceous plants maybe more adaptive to coldness by being annual or by the production of underground buds and stems (Latham and Ricklefs 1993; Donoghue 2008; Hawkins et al. 2011). In addition, habitat filtering may be strengthened with organism size, due to the fact that larger organisms are both less plastic in their fundamental niches and more able to be selective in dispersal (Aarssen et al. 2006; Farjalla et al. 2012).

### Patterns of phylogenetic structure on the Qinghai-Tibetan Plateau

The phylogenetic structure of ecological communities provides insights into the relative importance of different historical and ecological processes (Webb et al. 2008; Cardillo 2011). Historical effects such as higher *in situ* speciation, niche conservatism, and dispersal limitation may lead to phylogenetic clustering. By contrast, processes such as niche evolution, evolutionary convergence, and colonization may result in phylogenetic overdispersion in communities (Wiens and Donoghue 2004; Allen and Gillooly 2006). As for ecological processes, competitive exclusion and environmental filtering can both create nonrandom community phylogenetic structures (Cavender-Bares et al. 2006; Kraft et al. 2007; Webb et al. 2008). If environmental conditions in a habitat act as a filter that selects for species in possession of certain traits, we would expect phylogenetic clustering as closely related taxa are regarded to be ecologically more similar (Wiens and Graham 2004); whereas the pervasion of competition exclusion may result in phylogenetic dispersion, as interspecific competition is expected to be more intensive among more closely related taxa because of their similarity in resource requirements (Burns and Strauss 2011).

In this study, we observed positive NRI (phylogenetically clustering) across the plateau, excepting that it is negative (phylogenetically overdispersion) at the junction of eastern Himalaya and Hengduan Mountains at the southern edge of the plateau. We further observed that the NRI first kept constant and then decreased as mean annual temperature (MAT), monthly temperature of the coldest month (MTCM), and annual precipitation (AP) increased. These results suggest that the phylogenetic structure on the Qinghai-Tibetan Plateau clusters in most of the plateau, where the climate is cold and dry, and it tends to be overdispersed at the southern edge of the plateau where the climate tends to be warm and humid. Studies on the species or genus phylogeography show that the geographical isolation caused by the uplift of the Qinghai-Tibetan Plateau has promoted the *in situ* speciation on the plateau at the late Tertiary and led to the coexistence of closely related species (Yu and Zhang 2013). The Qinghai-Tibetan Plateau has experienced several glacial and interglacial periods during the Quaternary. Accordingly, although some cold- and drought-tolerant species may expand their distribution ranges, most species may shrink their distribution ranges, and some species may even migrate to shelters in lower and warmer places on the fringe of the plateau, in the glacial period (Yu and Zhang 2013). They may enlarge their distribution range in the interglacial periods. The uplift and oscillation in climate has created the severe environment on the plateau (Liu et al. 1999) that could act as a dispersion barrier and filtered out those vulnerable species at large scales (Cardillo 2011). On the other hand, huge mountains on the southeastern plateau may act as main shelters during the glacial period and provided a variety of habitats for the coexistence of distantly related plants. The joint effect of long-term geographical isolation caused by high mountains and deep valleys and interspecific competition has driven the divergence between sympatric species as well as exclusion of weaker competitors from their potential distributions (Cardillo 2011), thus led to the overdispersion phylogenetic structure in ideal environment.

Taken together, based on a comprehensive database on distributions of vascular plants on the Qinghai-Tibetan Plateau, we explored the patterns of diversity and the phylogenetic structure of vascular plants on the highest plateau on the earth. The results showed a strong association between species richness and climate, while the effect of habitat heterogeneity was insignificant. The relationship of species richness and climate remained even after the effect of habitat heterogeneity was controlled. However, the responses of richness to climate remarkably depended on life-forms. Richness of woody plants showed stronger climatic associations than that of herbaceous plants. The richness pattern of woody plants was regulated by energy and water availability together, whereas the richness of herbaceous plants was mainly regulated by water availability. From the phylogenetic perspective, rapid speciation and environmental filtering process together led to the assemblages of most communities on the plateau, and the lineages of vascular plants were quite clustered under severe climatic environments. Therefore, biodiversity conservation in this area should better take into account ecological features for different life-forms and phylogenetic lineages.
